# Detection and characterization of ESBL-producing *Enterobacteriaceae* from the gut of healthy chickens, *Gallus gallus domesticus* in rural Nepal: Dominance of CTX-M-15-non-ST131 *Escherichia coli* clones

**DOI:** 10.1371/journal.pone.0227725

**Published:** 2020-05-29

**Authors:** Supram Hosuru Subramanya, Indira Bairy, Niranjan Nayak, Rajesh Amberpet, Shashiraj Padukone, Yang Metok, Dharm Raj Bhatta, Brijesh Sathian

**Affiliations:** 1 Manipal College of Medical Sciences, Pokhara, Nepal; 2 Melaka Manipal Medical College, Manipal Academy of Higher Education, Manipal, India; 3 Jawaharlal Institute of Postgraduate Medical Education & Research, Pondicherry, India; 4 Bangalore Medical College and Research Institute, Bengaluru, India; Panstwowy Instytut Weterynaryjny - Panstwowy Instytut Badawczy w Pulawach, POLAND

## Abstract

The surge in the prevalence of drug-resistant bacteria in poultry is a global concern as it may pose an extended threat to humans and animal health. The present study aimed to investigate the colonization proportion of extended-spectrum β-lactamase (ESBL) and carbapenemase-producing *Enterobacteriaceae* (EPE and CPE, respectively) in the gut of healthy poultry, *Gallus gallus domesticus* in Kaski district of Western Nepal. Total, 113 pooled rectal swab specimens from 66 private household farms and 47 commercial poultry farms were collected by systematic random sampling from the Kaski district in western Nepal. Out of 113 pooled samples, 19 (28.8%) samples from 66 backyard farms, and 15 (31.9%) from 47 commercial broiler farms were positive for EPE. Of the 38 EPE strains isolated from 34 ESBL positive rectal swabs, 31(81.6%) were identified as *Escherichia coli*, five as *Klebsiella pneumoniae* (13.2%), and one each isolate of *Enterobacter* species and *Citrobacter* species (2.6%). Based on genotyping, 35/38 examined EPE strains (92.1%) were phylogroup-1 positive, and all these 35 strains (100%) had the CTX-M-15 gene and strains from phylogroup-2, and 9 were of CTX-M-2 and CTX-M-14, respectively. Among 38 ESBL positive isolates, 9 (23.7%) were Ambler class C (Amp C) co-producers, predominant were of DHA, followed by CIT genes. Two (6.5%) *E*. *coli* strains of ST131 belonged to clade C, rest 29/31 (93.5%) were non-ST131 *E*. *coli*. None of the isolates produced carbapenemase. Twenty isolates (52.6%) were in-vitro biofilm producers. Univariate analysis showed that the odd of ESBL carriage among commercial broilers were 1.160 times (95% CI 0.515, 2.613) higher than organically fed backyard flocks. This is the first study in Nepal, demonstrating the EPE colonization proportion, genotypes, and prevalence of high-risk clone *E*. *coli* ST131 among gut flora of healthy poultry. Our data indicated that CTX-M-15 was the most prevalent ESBL enzyme, mainly associated with *E*. *coli* belonging to non-ST131clones and the absence of carbapenemases.

## Introduction

The family *Enterobacteriaceae* exhibit antimicrobial resistance mainly due to the production of extended-spectrum β-lactamases (ESBL), AmpC β-lactamases, and carbapenemases [1]. Superbugs that are resistant to multiple classes of antibiotics like tetracycline, fluoroquinolone, trimethoprim owing to the acquisition of ESBL genes have increased rapidly worldwide. Food-producing animals are considered to be an important reservoir of various multidrug-resistant (MDR) microbes [2]. Animal-derived *Enterobacteriaceae*, particularly *Escherichia coli*, can be pathogenic to humans and may act as a donor of resistance genes to other pathogens. Among ESBL genes, CTX-M-15 producing *E*. *coli* sequence type 131 (ST131) is a well-established pandemic clone causing significant extraintestinal infections in humans [3]. Poultry and other food-producing animals harboring *E*. *coli* ST131 clones can spread to humans either directly via consumption or through environmental pathways. The emergence and rapid spread of ESBLs’ among *Enterobacteriaceae* is a major hurdle in treating severe infections of livestock as well as humans [4].

Gut colonization by extended-spectrum β-lactamase-producing *Enterobacteriaceae* (EPE) and carbapenemase-producing *Enterobacteriaceae* (CPE) is an essential factor for the spread of ESBL bacteria among poultry and other livestock [1, 2, 4]. During the last decade, EPE from a different variety of food-producing animals like cattle, poultry, and pigs have been documented worldwide [5–10]. Several studies have found that poultry meat and poultry products carried the highest contamination with ESBL-producing bacteria [7]. Inappropriate use of antibiotics in human and animals have resulted in the global emergence of *E*. *coli* clone ST131, which is resistant to both fluoroquinolones and extended-spectrum cephalosporins due to the production of the ESBL CTX-M-15 [11]. The plausibility of this clone being able to acquire plasmids encoding carbapenemases in the future is a matter of significant concern. A few studies have even reported the presence of carbapenemase-producing bacteria in animals [8]. An accurate picture of the extent of widespread ESBLs among poultry will be important in determining the significance of these reservoirs as potential sources of transmission to humans. It is well-known that endogenous flora of poultry origin can spread via the food chain and transiently colonize the human gut [12, 13]. It is, therefore, crucial to identify and reduce a load of EPE and CPE colonization in poultry birds.

In Nepal, 3.5% of the national Gross domestic product (GDP Per Capita 1,034.118 USD, 2019) is contributed by poultry farming, and poultry meat is the most common meat source [14]. More than 64 districts in the country are involved in conventional poultry farming, of which 93.29% produce broilers. Around 51.9% of households in Nepal are involved in backyard poultry production by the scavenging and semi-scavenging system; the most substantial proportion is found in the Hills (67.8%), followed by Mountain (56.4%) and Terai regions of Nepal [14].

The prevalence of EPE and CPE and their clonal diversity among gut flora of poultry vary globally. At present, there is no data available from Nepal. The present study aimed to investigate the colonization proportion of EPE and CPE in the gut of healthy poultry, *Gallus gallus domesticus* in Kaski district, western Nepal. We also compared the rate and load of EPE isolates between healthy organically-raised backyard chickens and commercially grown chickens. Further, we investigated the distribution of ESBL genes, pandemic *E*. *coli* clonal group ST131 and co-resistance among the EPE isolates.

## Material and methods

### Study design, the site, and enrollment of farms

During the study period (October 2016 to January 2018), districts in Nepal were divided into political units called Village Development Committees (VDCs), and the metropolitan regions were divided into wards. Nepal was divided into 14 zones. The study area Kaski district is under the Gandaki zone. The sample size was determined based on prevalence rate, as reported by the previous study by Hussain et al. [15]. We applied the cluster random sampling method. All 47 VDCs of Kaski district was numbered based on alphabetic order, 23 (49%) VDCs were selected by random sample calculation using formula = RANDBETWEEN (1, 47). The random numbers generated were 1, 2, 4, 15, 40, 27, 16, 33, 31, 5, 22, 17, 37, 26, 45, 34, 23, 9, 41, 39, 8, 13, and 28. A total of 113 rectal swabs specimens from poultry birds were collected from 113 farms (66 backyard and 47 commercial farms) distributed over the Kaski district in western Nepal (S1 Fig). On average, two commercial farms and two backyard farms were selected per VDC. The region is inhabited by rural folk of low socioeconomic backgrounds living with poor hygiene. Selection of the farms for our study was based on their geographical location (rural region), age of the birds (preferably middle-aged), type of the breed (broiler-type among commercial poultry), preferably with 100 chickens per breeding site in case of commercial farms and ten birds in case of the backyard farms), adequate husbandry practice and veterinary intervention information obtained from the regional veterinarians. Backyard chicken farms that were near (less than a mile) to commercial poultry farms were not included in this study. Randomly, two birds from the backyard and five from commercial farms were sampled from each selected farm. The commercial poultry farms were of broiler-type, which were caged, and fed commercially available poultry feeds. Backyard breeds included local/ indigenous breeds of *G*. *domesticus* (Sakini, Ghanti Khuile) and Giriraja, the Indian breed, which is commonly used for backyard farming. These were raised as free-roaming birds and were fed with locally available organic food like rice, wheat, and other grains. All the investigated birds were healthy with a residency of at least 15 days among broilers and a month among backyard chickens. None of the broilers had been treated with any medications, for at least two weeks before sampling. Backyard chickens were organically-raised and had no history of direct exposure to antibiotics or any other medication.

### Specimen collection and questionnaires

Two birds from each backyard and five from each commercial farm were randomly selected for sampling. Swabs collected from the individual farm were pooled and processed as one specimen for further microbiological investigation. A sterile cotton swab pre-moistened with sterile normal saline was inserted into the chicken rectum (1–1.5 inches deep) and gently rotated. The swabs were placed in a sterile tube and transported to the research laboratory, Manipal Teaching Hospital, Nepal, in specimen transport containers with ice packs. The samples were processed within 8 hours of collection. Detailed questionnaires, including demographic and clinical characteristics, were filled up using appropriate pro forma (S1 File).

### Sample processing and screening for EPE

The rectal swab was placed in 1 ml of sterile 0.9% saline and vortexed for 30 seconds. For performing viable bacterial count, a serial tenfold dilution (10^−1^ to 10^−4^) was first carried out, following which a small amount of suspension (100μL) was cultured on commercial ESBL-selective chromogenic medium (HiMedia, India) for screening EPE and that of carbapenem-resistant *Enterobacteriaceae* on MacConkey agar with 1 μg/mL imipenem (HiMedia, India). Plates were incubated at 37°C under aerobic conditions and assessed after 24 hours of incubation. For the presumptive identification of EPE isolates, the color of the colonies was recorded according to the color chart provided by the manufacturer. A single isolated colony of each color was picked and subcultured on nutrient agar, was used for phenotypic identification and preservation. Isolates were speciated by standard phenotypic methods [16]. All ESBL positive isolates were preserved in brain heart infusion-glycerol broth in stock vials and stored at -20°C for further study.

### Antibiotic susceptibility testing

The antibiotic susceptibility profiles of the ESBL-screen positive isolates were determined after testing against a panel of fifteen antibiotics of human and veterinary clinical relevance by Kirby-Bauer disk diffusion method was performed as per CLSI guidelines and interpreted based on CLSI 2017 and 2018 breakpoints [17, 18]. Susceptibility to tigecycline was interpreted with FDA breakpoints (http://www.accessdata.fda.gov/drugsatfda_docs/label/2009/021821s016lbl.pdf). Included were one or two representatives from various classes of antibiotics: cefotaxime and ceftazidime (3^rd^ generation cephalosporins), cefoxitin (2^nd^ generation cephalosporins), co-amoxiclav (beta-lactam combination agents), tetracycline (tetracyclines), gentamicin, and amikacin (aminoglycosides), nalidixic acid (synthetic quinolone), norfloxacin, and ciprofloxacin (fluoroquinolones), nitrofurantoin (nitrofurans), Co-trimoxazole (folate pathway inhibitors), chloramphenicol (amphenicol), tigecycline (glycylcycline), and imipenem, meropenem and ertapenem (carbapenems). *E*. *coli* ATCC 25922 (negative control) and *K*. *pneumoniae* ATCC 700603 (positive control) were used for quality control. Antimicrobial disks used were obtained from Mast (MASTDISCS^™^), UK.

### ESBL confirmation

#### Phenotypic methods

*The double-disc synergy test*. The isolates grown on ESBL HiChrome agar were retested for ESBL production by the Double Disc Synergy Test (DDST) [18]. Pairs of discs containing extended-spectrum cephalosporin [cefotaxime (30 μg) and ceftazidime (30μg) alone and with clavulanic acid (10 μg) were placed 25 mm apart, center to center, on a Mueller Hinton Agar (MHA) plates inoculated with a bacterial suspension matching with 0.5 McFarland turbidity. After overnight incubation at 37°C, zone diameters were measured. ESBL-producing isolates were defined as strains resistant to cefotaxime (zone diameter ≤27 mm) or ceftazidime (zone diameter ≤22mm), and an increase in zone diameter ≥ 5mm with the disks containing clavulanic acid. S2 Fig.

*AmpC detection*. All the ESBL positive isolates, which showed resistance to cefoxitin in the initial screening, were confirmed for the AmpC enzyme co-production by saline disc method and inhibitor-based tests [19, 20] S2 Fig.

*Phenotypic screening of carbapenemases*. All ESBL isolates were screened phenotypically for carbapenemase production by modified Hodge Test [21], and modified carbapenem inactivation method [22].

*In-vitro biofilm formation assay*. Biofilm formation was performed following the Xu Z. et al. [23] with slight modification. The exponential growth phase of bacteria was incubated at 37°C for 48 hours in 96-well microtiter plates. Non-adherent cells were removed by washing the biofilms with phosphate buffer saline (PBS) and stained with crystal violet solution. The absorbance was measured at 595nm after de-staining the biofilm with 99.8% ethanol. The assay was done in duplicates with the known positive and negative control. The biofilm formation was determined by calculating the cut-off values from the arithmetic mean of the absorbance of negative controls with three times the addition of standard deviation.

*Molecular characterization of β-lactamase encoding genes*. Isolates displaying an ESBL phenotype were screened for ESBL encoding genes blaC_TX-M_, including phylogenetic groups 1, 2, and 9 by multiplex PCR as described by Dallenne C, et al. [24]. The presence of β-lactamase genes bla_TEM_/bla_SHV_/bla_OXA_ was also detected by multiplex PCR assays [24]. The primers (Sigma-Aldrich) used, and the size of the expected DNA products for each enzyme group are shown in S1 Table. DNA extraction was performed by a commercially available DNA purification kit (HipurA bacterial genomic DNA purification Kit, Himedia, India). PCR reactions were carried out in 25 μL volumes. This included 2 X Amplicon Red Taq master mixes consisting of Tris HCl pH 8.5, (NH4)2 SO4, 4 mM MgCl2, 0.2% Tween 20, 0.4 mM dNTPs, and 0.2 units/μL amplicon Taq DNA polymerase. The primers were used between 0.2μM to 0.4 μM concentrations. The template DNA was used in 1μL volume (S2 Table). Cycling protocol was as follows: initial denaturation at 94°C for 10 min; 30 cycles of 94°C for 40 s, 60°C for 40 seconds and 72°C for 1 min; and a final elongation step at 72°C for 7 min.

Isolates positive for phylogroup-1 were further characterized for blaCTX-M 15 genes by uniplex PCR assay [25]. Amplification was performed with 2X Amplicon Red Taq master mixes (10 μL); the 1μL volume of primer (forward and reverse) and 1μL of purified DNA in a total volume of 25 μL. Amplicons were visualized after running at 100V for one hour on 2% agarose gel containing ethidium bromide. The ESBL subtypes were further confirmed by sequencing. Obtained sequences were aligned using Bioedit V.7.2.1 software, and consensus sequences were compared against the sequences deposited in the Genbank database with the help of the Basic Local Alignment Search Tool (BLAST) program (http://www.ncbi.nlm.nih.gov/BLAST).

*AmpC genes*. The presence of plasmid-mediated AmpC genotypes FOX (FOX-1 to FOX-5, MOX-1), MOX (MOX-2, CMY-1, CMY-8 to CMY-11 and CMY-19), DHA (DHA-1 and DHA-2), CIT (LAT-1 to LAT-3, BIL-1, CMY-2 to CMY-7, CMY-12 to CMY-18 and CMY-21 to CMY-23) were determined by multiplex nucleic acid amplification assay. The primers and PCR conditions were followed, as described by Dallenne C et al. [24]. The amplification products were confirmed by agarose gel electrophoresis.

*E*. *coli ST131 clade PCR assay*. All ESBL positive *E*. *coli* isolates were screened for sequence type 131 clonal lineage and ST131 clades (A, B, and C) by multiplex PCR as described by Matsumura Y, et al. [26]. Amplicons were visualized after running at 100 V for one hour on 2% agarose gel containing ethidium bromide. A 100 bp DNA ladder (Eurofins Scientific, India) was used as a size marker. Known *E*. *coli* ST 131 and non-ST 131 *E*. *coli* were used as positive and negative controls, respectively (S3 Fig). The primers used in this study and the size of the expected DNA products for each enzyme group is listed in S1 Table.

### Statistical analysis

The Epi-info and SPSS software were used to assess the significant differences in the prevalence and high degree of antibiotic resistance between the different populations. GraphPad Prism Version 8.1.2 (227) was used to generate a heat map showing an antibiotic resistance profile and ESBL genes.

#### Ethics approval and consent to participate

The research proposal was approved by the Institutional Ethics Committee, Manipal Teaching Hospital, Pokhara, Nepal. Reference number: MEMG/IRC/GA30/11/2014.

## Result

### Rate of ESBL-E in commercial broilers and backyard chickens

Out of 113 pooled rectal samples obtained, 28.8% specimens (19/66) from backyard farms and 31.9% (15/47) from commercial farms grew EPE. None of the isolates was carbapenemase producer by phenotypic tests. Of the 38 EPE strains (22 from the backyard farms and 16 from commercial farms) isolated from 34 specimens, 31(81.57%) were identified as *Escherichia coli*, five as *Klebsiella pneumoniae* (13.1%), and one each isolate as *Enterobacter* species and *Citrobacter* species (2.6%). Among 38 ESBL positive isolates, 09 (23.6%) were AmpC co-producers. Median age (IQR) of ESBL positive chickens were 75.5 days (48.5, 157.5) and ESBL negative was of 60 days (44, 120). Univariate analysis showed that among the commercial chickens, the odd of ESBL carriage is 1.160 times (95% CI, 0.515, 2.613) higher compared to backyard birds (Table 1). Besides, the univariate analysis also showed that there was a 1.1% increase in the likelihood of EPE colonization rate in backyard birds according to one unit increase in age [odds ratio 1.011, (1.003, 1.020), p = 0.01]. However, there was a 1.3% decrease in the likelihood of EPE colonization rate in commercial chickens according to one unit increase in age [odds ratio 0.987, (0.956, 1.020), p = 0.4].

**Table 1:** Details of the commercial and backyard poultry and the organisms isolated in each group.

	Total number (%)	Backyard chickens	Commercial chicken	P-value
**Total**	113	66 (58.4%)	47 (41.6%)	-
**ESBL negative samples (%)**	79 (69.9%)	47 (71.2)	32 (68.1)	0.721
**ESBL positive samples (%)**	34 (30.1%)	19 (28.8%)	15 (31.9%)	
**Colony count CFU/mL**				
Geometric Mean	5×10^6^	1.5×10^6^	2.9×10^7^	--
Range	3×10^2^–8.7×10^9^	2.8×10^4^–4.9×10^7^	3×10^2^–8.7×10^9^	
**Carbapenemase**	00 (00)	00 (00)	00 (00)	--
**ESBL + AmpC positive (%)**	9 (7.9%)	5 (7.6)	4 (8.5)	--
**Age**				
Median Age (IQR) days	65 [44,120]	120 [60, 150]	44 [32, 60]	**--**
**Feed**				
Organic feed	--	66 (58.4%)	0	
Commercial feed		0	47 (41.6%)	**--**
**Isolates**	38	22	16	**--**
*E*. *coli*	31 (81.6)	16 (72.7)	15 (93.7)	0.09
*Klebsiella pneumoniae*	05 (13.1)	04 (18.2)	1 (6.5)	0.28
*Enterobacter* species	01 (2.6)	01 (4.5)	00	--
*Citrobacter* species	01 (2.6)	01 (4.5)	00	--

#### Concentrations of EPE in rectal swabs

The concentrations of EPE among examined poultry birds ranged between 3×10^2^–8.7×10^9^ CFU/mL with a geometric mean 5×10^6^ CFU/mL (Table 1). The proportion of ESBL-producing bacteria was slightly higher among commercial chickens as compared to backyard chickens; colony count (geometric means) was 2.9×10^7^ CFU/mL, and 1.5×10^6^ CFU/mL, respectively. This difference, however, was not statistically significant (P>0.1).

### Diversity among β-lactamase genes

**Fig 1:**
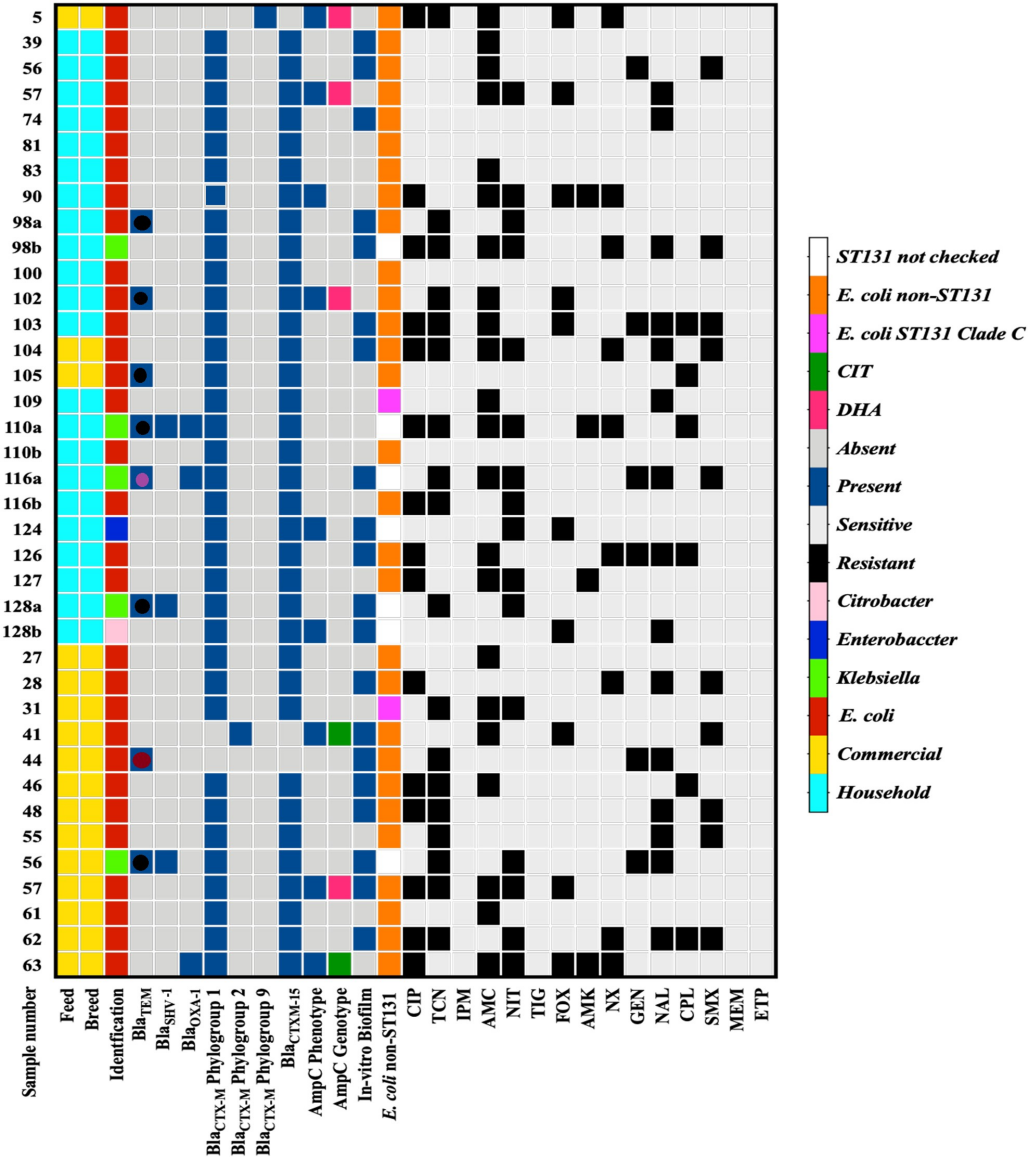
Antibiotic susceptibility and β-lactamase genes identified in 38 EPE strains isolated from commercial and backyard chickens. **blaTEM**: bright red circle = bla_TEM-29_, black circle = bla_TEM-1_, Pink circle = bla_TEM-2_. **CIP**: ciprofloxacin, **TCN**: tetracycline, **IPM**: imipenem, **AMC**: co-amoxiclav, **NIT**: nitrofurantoin, **TIG**: tigecycline, **FOX**: cefoxitin, **AMK**: amikacin, **NX**: norfloxacin, **GEN**: gentamicin, **NAL**: nalidixic acid, **CPL**: chloramphenicol, **SMX**: Co-trimoxazole, **MEM**: meropenem, **ETP**: ertapenem.

All thirty-eight isolates phenotypically positive for ESBL production was subjected to genotypic characterization via multiplex PCR assay. Twenty-one percent (8/38) of the strains co-harbored multiple β-lactamase genes, with the most common being the combination of *bla*_CTX-M-15_ and *bla*_TEM-1_. The *bla*_TEM_ β-lactamase gene was detected in 8 (21.1%) isolates, out of which six *bla*_TEM-1_ and one *bla*_TEM-2_ positive isolates were also carrying the bla_CTX-M-15_ gene and rest one isolate was carrying *bla*_TEM-29_ type alone. *bla*_*SHV-1*_ and *bla*_OXA-1_ genes were detected in 3 (7.9%) isolates each (all six strains were co-harbored *bla*_CTX-M-15_ genes), Fig 1, and S2 Table. Among *bla*_CTX-M_ type ESBLs, phylogenetic group-1 (variants of CTX-M group 1) was found in 35 (92.1%) EPE isolates, phylogenetic group-9 (variants of CTX-M group 9) and phylogenetic group-2 (variants of CTX-M group 2) were found in one (2.63%) isolate each. Further characterization of phylogroups revealed that all 35 (100%) phylogroup-1 positive EPE strains were of CTX-M-15 types and isolate from phylogroup-2, and 9 were of CTX-M-2 and CTX-M-14 respectively. There was no significant difference in the distribution of ESBL genes among commercial and backyard chickens. However, *bla*_CTX-M-15_ genes were detected in 100% (22/22) of EPE strains isolated from backyard chickens and that of 81.3% (13/16) from commercial chickens.

Among nine phenotypic AmpC positive isolates, four were positive for the DHA gene (DHA-1 and DHA-2), and two isolates were positive for the CIT gene (LAT-1 to LAT-3, BIL-1, CMY-2 to CMY-7, CMY-12 to CMY-18 and CMY-21 to CMY-23), the rest of the three isolates were negative.

### Antibiotic resistance profile

Antibiotic sensitivity/resistance data obtained after the standard disc diffusion tests are shown in Fig 1. EPE isolates of broiler chickens showed the highest resistance towards tetracycline (62.5%) followed by amoxicillin (56.3%) and ciprofloxacin (50%). The isolates of backyard chickens were relatively lower resistance towards tetracycline (36.4%), ciprofloxacin (31.8%), but higher resistance against amoxicillin (56.5%) and nitrofurantoin (59.1%). 25% of the isolates of commercial chickens and that of 27.3% of backyard chickens showed resistance to cefoxitin. Resistance against nalidixic acid was 36.4% among isolates of backyards and 43.8% among commercial chickens. Each of 18.2% of backyards’ isolates while 12.5% and 37.5% of broilers were resistant to gentamicin and cotrimoxazole. Both the isolates of commercial and backyards’ chickens showed lower resistance to amikacin (13.6% and 6.3%, respectively). All of the ESBL producing strains were susceptible to imipenem, meropenem, ertapenem, and tigecycline.

#### Biofilm production

Twenty EPE isolates (20/38, 52.6%) were invitro-biofilm producers. Out of 20 biofilm-producing phenotypes, 56.5% (9/16) were from commercial, and 50% (11/22) were of backyard chicken (Fig 1). However, within the 20 isolates, 9 of them were weakly adherent, 11 were moderately adherent, while none of the EPE isolates exhibited high capability for biofilm production.

#### ST-131 clones among *E*. *coli*

Two (6.5%) *E*. *coli* strains of ST131 belonged to clade C, 29/31 (93.5%) were non-ST131 *E*. *coli*. Both the *E*. *coli* ST 131 clade C strains were found among commercial chickens (Fig 1 and S3 Fig).

## Discussion

Misuse of antibiotics has been proposed as one of the significant factors for the selection and emergence of resistant strains both in veterinary and clinical medicine [27, 28]. The quantity of antibiotics used in animal husbandry often exceeds their use in medical practice [29]. Antibiotic-mediated selection of resistance not only happens in pathogenic bacteria but also among the normal bacterial flora of exposed individuals (animals and/or humans) or populations [28, 30, 31]. There can also be the possibility of continuous feeding of antimicrobial agents to food animals like broilers in the form of antimicrobial growth promoters/non-therapeutic antibiotics or other preventive usages like inovo medication or usage of zinc bacitracin. Usually, commercially available poultry feed (formulated feed) has low doses of antibiotics which are taught to be must be needed to promote fast growth and expedite weight gain [32].

Generally, crowding and poor sanitation play an important role in the selection of resistance. Hence, the development of drug resistance among bacteria in broiler chickens due to high antibiotic selection pressure may constitute a high proportion of resistant bacteria in their fecal flora [33]. These factors are typical of intensive commercial poultry farming and explain the high rate of resistance in fecal flora of broilers, as shown in the present study and studies carried out previously [34, 35]. Although zoonotic transmission of ESBLs from animals to humans or vice-versa remains controversial, several studies showed a direct link like transmission through intimate contact with the animals or via undercooked/uncooked meat handling/consumption [35]. Olaitan et al. demonstrated the zoonotic transmission of a colistin-resistant gene *mcr-1* from a pig to its rearer [36].

Global studies reported a wide range of prevalence of EPE among commercially grown poultry flocks. Previous studies showed a 0.7% prevalence of EPE among broiler chickens in France, 44% in Germany, 60% in Japan, and that of 81% in the Netherlands [37, 38]. Similarly, EPE prevalence was 69%, 60%, 20.6%, and 3.2% among commercially grown poultry flocks in Romania, Ecuador, Lebanese, and Vietnam [39]. A study conducted by A. Shrestha et al. [40] in the Bharatpur metropolitan, Nepal, had reported 36.9% ESBL positive bacterial contamination in chicken meat samples. Likewise, M. Subedi et al. [41] reported a high prevalence of MDR *E*. *coli* and high frequency of virulence genes in avian pathogenic *E*. *coli* strains isolated from the colibacillosis suspected broiler chickens in Chitwan, Nepal. In Nepal, the prevalence of EPE colonization in the gut of poultry birds has not been studied so far. This study is the first epidemiological study in Nepal quantifying the rate of intestinal carriage of ESBL genes among *Enterobacteriaceae* in healthy chicken flocks. This study is unique in having evaluated local backyard/organic-raised chickens for such colonization, and we found 28.8% and 31.9% prevalence rate of EPE in the backyard chickens and commercial chickens, respectively.

The local breed of backyard chickens sampled in this study had no direct exposure to antibiotics. They are organically fed by homegrown cereal, and often they are naturally resistant to common infections. These birds were rarely exposed to antibiotic treatment. Interestingly, the results of this study suggested a high rate of EPE both in commercial broilers and domesticated backyard chickens.

High rate of EPE was reported previously by JC Stuart and colleagues from Netherland [42], and A Kola and colleagues from Germany [43], in both conventional and organic poultry meat with several strains having similar sequence type to those reported from human origin.

The high rate of gut colonization of EPE in organically-raised backyard chickens is surprising, considering the strict limitation of antibiotic usage. Very few studies in the past noted similar findings [44]. In developing countries like Nepal, with inadequate protection of human and animal excreta and water, contamination of the environment with antibiotic-resistant bacteria might appear to play a great role in EPE carriage amongst backyard chickens. Another possible explanation for the high rate of detection of EPE in backyard chickens may be due to rampant use of antibiotics in human and/or animal populations, the risk of carrying resistant organisms caused by household members’ use of antibiotics, interfamilial transmission, or proximity to an antibiotics source [45]. All the backyard chickens involved in this study were free-roaming. Being good natural scavengers, these flocks might feed on a variety of insects and also green foliage, farm, and kitchen waste. Therefore, the possible transmission of resistant bacteria from the above sources cannot be ruled out [46]. The prevalence of ESBL genes among backyard chickens could also suggest that the antimicrobial resistance could be acquired by multiple sources like environment, contacts, and not necessarily by the personal antibiotic consumption alone [47].

In the present study, *Escherichia coli* were the major bacterial species among EPE, a finding similar to global reports [48]. Molecular characterization revealed that 92.1% of strains harbored *bla*_CTX-M-15_ genes. It can be explained by supportive evidence from earlier meta-analysis suggesting globalization of *bla*_CTX-M_ type EPE, with a high rate of dissemination of *bla*_CTX-M-15_ types in the Asian subcontinent [47, 48]. In this study, Non-β lactam co-resistance in ESBL and/or AmpC co-producers toward tetracycline and ciprofloxacin was as high as 62.5% and 50% each among broilers while relatively low among backyard chickens (36.4% and 31.8% respectively). It is possibly linked to the frequent use of these antibiotics in animal fodder and veterinary medicine [8, 11, 30].

The study conducted by Nikolay PB et al. [49] in Northwestern Ecuador, found that production birds, including broilers and layers, had high levels of antibiotic resistance and a notably higher proportion of resistant isolates than household birds. They also observed the prevalence of resistant phenotypes tended to decrease with bird age for drugs like ampicillin, amoxicillin/clavulanate, cefotaxime, cephalothin, chloramphenicol, ciprofloxacin, gentamicin, and streptomycin (*P* < 0.05) except sulfisoxazole, trimethoprim-sulfamethoxazole, and tetracycline. A similar trend we observed in our study, that among broiler, the EPE colonization rate tends to decrease with the birds' age (0.987, (0.956, 1.020), p = 0.4). In contrary to that, among backyard chickens, the EPE colonization rate tends to increase with the birds' age (OR 1.011, (1.003, 1.020), p = 0.01).

In the present study, more than 50% of EPE isolates of both commercial and backyard chickens possess a biofilm-producing phenotype. *E*. *coli* is one of many bacteria that can switch between planktonic form and biofilm form. Biofilm cells are about 1000 times more resistant than their planktonic form [50]. Biofilm producing EPE strains are recalcitrant to immune factors and antibiotic therapy and are often responsible for chronic infection, which is extremely resistant to treatment. *E*. *coli* ST131 can attain a combination of successful spread, antibiotic resistance, and virulence. Only a few studies have investigated the presence of *E*. *coli* ST131 in food animals. *E*. *coli* ST131 isolates producing CTX-M-9 have occasionally been recovered from poultry feces. In some of these instances, the animal isolates have presented a certain similarity to human ST131 isolates [51, 52]. Concurrent to our study, non-ST131 isolates were identified by Wu et al. and Randall et al. al among chickens [53, 54] and prevalence rate of 7% *E*. *coli* ST131 was isolated from chicken meat samples [40]. All *E*. *coli* ST131 strains in our study belonged to clade C. Since, *E*. *coli* ST131 clade C adapt to environmental changes rapidly than other extraintestinal pathogenic *E*. *coli*, it is an utmost important to stop its continuing spread to gain next clonal wave of MDR extraintestinal pathogenic *E*. *coli*.

The risks to human health posed by EPE from non-human reservoirs are not fully understood. Surveillance for ESBL-producing bacteria in poultry could provide information on the fecal flora, which may contaminate the products entering the food chain. Especially in developing countries like Nepal, a multi-disciplinary approach involving public-private partners, government agencies are needed to limit the risk of AMR and minimize their impact on human and animal health [55]. The interventions like immunization, public awareness, upgraded sanitation practices, antibiotic stewardship, reduced use of antibiotics in agriculture, and livestock need to be implemented. Consequently, we believe that collecting local epidemiological data will help to shape future intervention strategies to reduce the spread of antibiotic-resistant strains and its negative consequences.

### Limitation and recommendation

Though the study is foremost in Nepal, it has few shortcomings. All PCR products were not subjected to sequencing to further identify the ESBL types. Similarly, carbapenemase genes were not amplified by genotype-based techniques. Also, this study lacks the detailed investigation of different sequence types and clonal diversity of organically raised backyard chickens and commercial broiler chickens. Therefore, further large scale studies, including intense targeted surveillance, molecular typing, and genomics-based analysis such as next-generation sequencing, would help to understand the spread and association of EPE in human and animal infections. Recently, Wielders CH and colleagues [56] observed that there is a seasonal variation of EPE colonization in the Dutch population. Therefore the seasonal colonization rate detection of EPE and CPE for poultry should be taken into account while designing future prevalence studies, especially in temperate regions.

## Conclusion

This is the first study in Nepal, demonstrating the EPE colonization rate, genotypes, and clonal prevalence among gut flora of healthy poultry, *Gallus gallus domesticus*. Our data indicate that CTX-M-15 was the most prevalent ESBL enzyme, mainly associated with *E*. *coli* belonging to non-ST131clones and the absence of detection of carbapenemases. This study highlighted that healthy organically raised backyard chickens in rural areas equally serve as potential reservoirs of ESBL producing *Enterobacteriaceae* as that of commercial broiler chickens. A further large-scale study is warranted to identify the significant predisposing factors for gut colonization by ESBLs and to recognize and monitor the associated risks due to such colonization in poultry birds.

## Supporting information

S1 Fig Location of Kaski District of Nepal where samples were collected (saffron color). https://commons.wikimedia.org/wiki/File:Political_Map_of_Kaski_District.jpg.
(TIF)

S2 Fig **A**–The double-disc synergy test- Ceftazidime disc (30 μg) with zone diameter ≤22mm and an increase in the inhibition zone diameter of >5 mm for ceftazidime + clavulanic acid (30 μg/10 μg) versus ceftazidime disc (30 μg) alone confirmed ESBL production (Fig 1). **B**–Saline disk Test -A positive AmpC test appeared as a flattening or indentation of the cefoxitin inhibition zone in the vicinity of the test organism. A negative test had an undistorted zone.
(PNG)

S3 Fig Agarose gel electrophoresis of the CTXM-15 type ESBL genes (A) and *E*. *coli* ST-131 clade (B). **A**. Lane M, 100-bp DNA ladder; lane 1- Positive control (known CTXM-15 positive isolate); lane 2–10 test strain positive for CTXM-15 genes (996bp); lane 11- negative control (normal saline). **B**. Agarose gel electrophoresis of *E*. *coli* ST131 clade PCR: Lane M, 100-bp DNA ladder; lane 1- negative control (normal saline), lane 2- positive control; lane 3- test strain non-ST131 *E*.*coli*, lane 3- test strain *E*.*coli* ST131 clade C (the resultant image is a product of time-averaged data).
(PNG)

S1 Table List of primers. (DOCX)

S2 Table Summary of ESBL genes identified in 38 ESBL producing isolates. (DOCX)

S1 File Questionnaires. (DOCX)

## Abbreviations

Amp C: Ambler class C
ATCC: American Type Culture Collection
CFU/mL: colony-forming units per milliliter
CLSI: The Clinical & Laboratory Standards Institute
CRE: Carbapenem-resistant *Enterobacteriaceae*
DDST: Double Disc Synergy Test
EPE: ESBL producing *Enterobacteriaceae*
GM: geometric mean
IQR: interquartile range
