# Supplementary figures and images for: Detection and characterization of ESBL-producing Enterobacteriaceae from the gut of healthy chickens, Gallus gallus domesticus in rural Nepal: Dominance of CTX-M-15-non-ST131 Escherichia coli clones
Source: PLoS One. 2020 May 29;15(5):e0227725. doi: 10.1371/journal.pone.0227725 (PMC7259619; doi:10.1371/journal.pone.0227725)

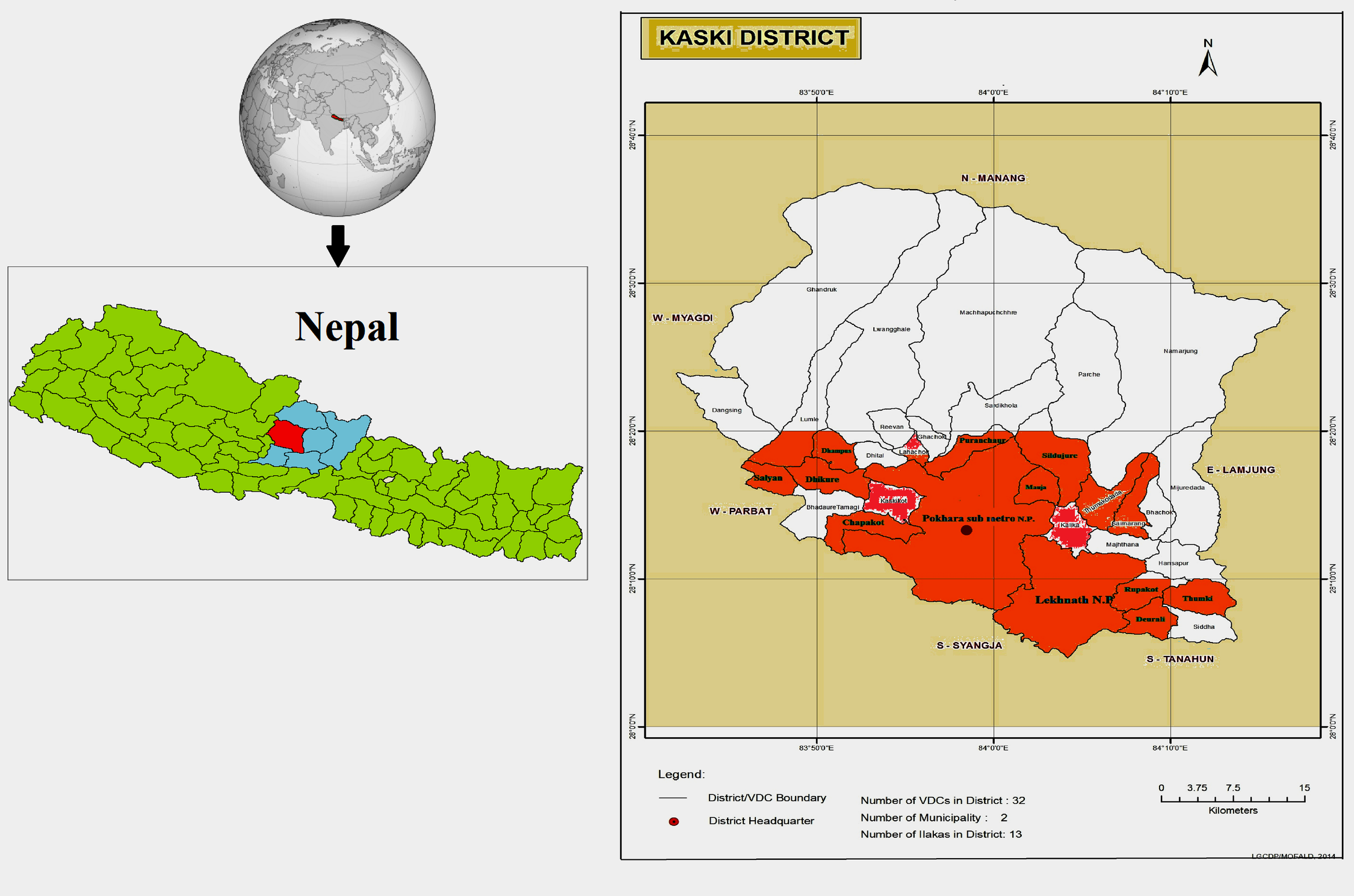

Supplement: S1 Fig — https://commons.wikimedia.org/wiki/File:Political_Map_of_Kaski_District.jpg. (TIF) [file pone.0227725.s001.tif]

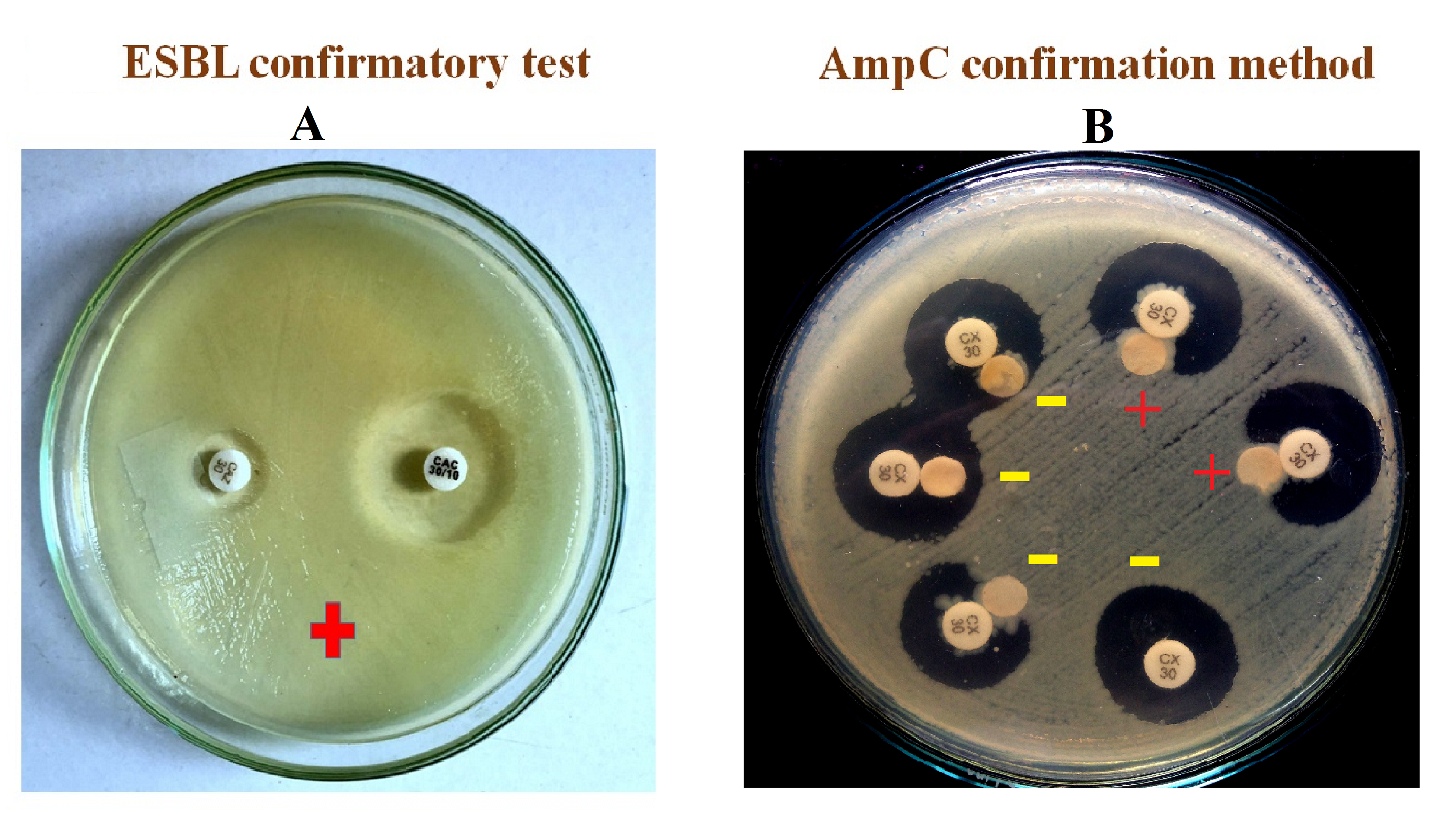

Supplement: S2 Fig — A–The double-disc synergy test- Ceftazidime disc (30 μg) with zone diameter ≤22mm and an increase in the inhibition zone diameter of >5 mm for ceftazidime + clavulanic acid (30 μg/10 μg) versus ceftazidime disc (30 μg) alone confirmed ESBL production (Fig 1). B–Saline disk Test -A positive AmpC test appeared as a flattening or indentation of the cefoxitin inhibition zone in the vicinity of the test organism. A negative test had an undistorted zone. (PNG) [file pone.0227725.s002.png]

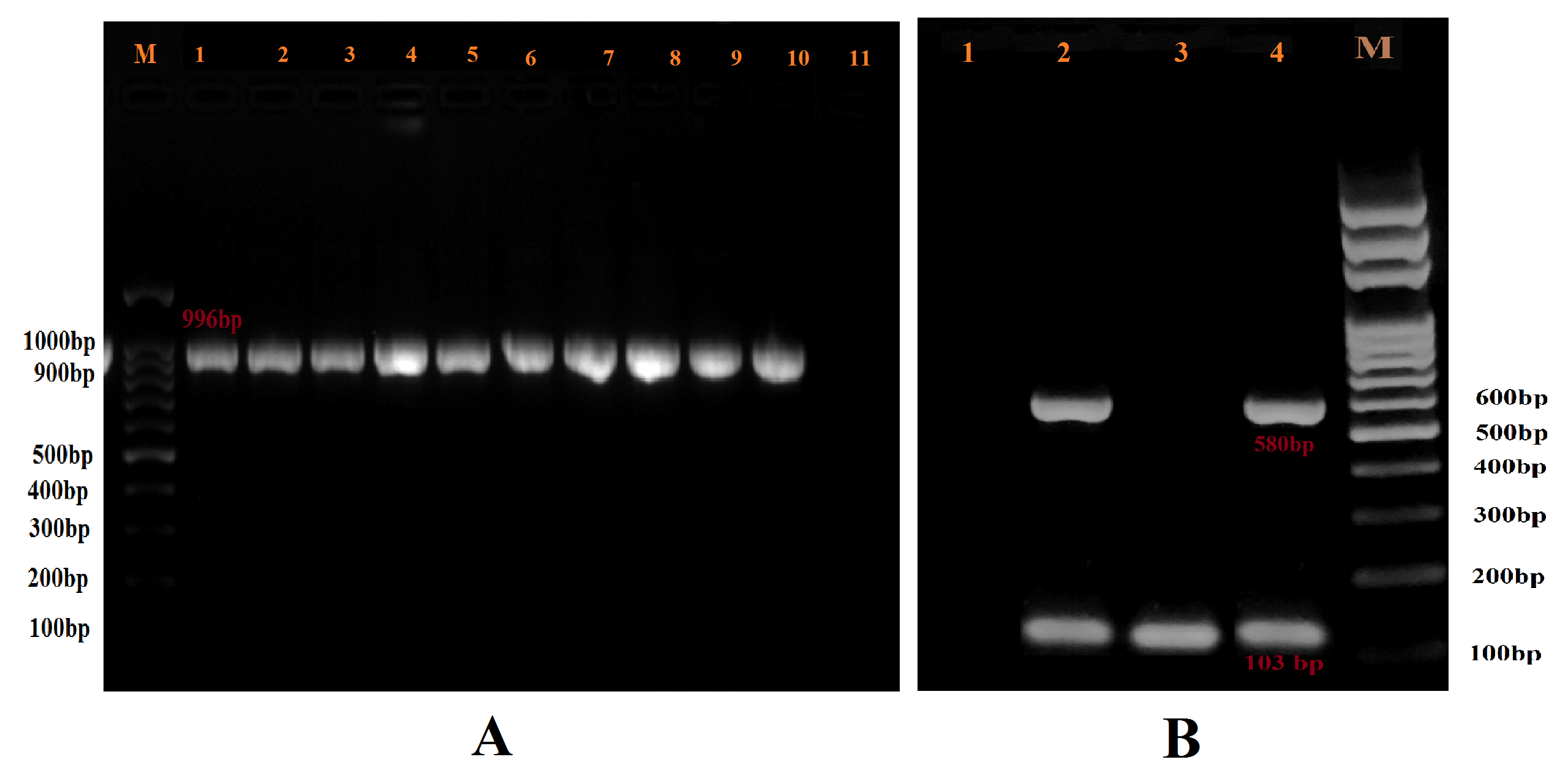

Supplement: S3 Fig — A. Lane M, 100-bp DNA ladder; lane 1- Positive control (known CTXM-15 positive isolate); lane 2–10 test strain positive for CTXM-15 genes (996bp); lane 11- negative control (normal saline). B. Agarose gel electrophoresis of E. coli ST131 clade PCR: Lane M, 100-bp DNA ladder; lane 1- negative control (normal saline), lane 2- positive control; lane 3- test strain non-ST131 E.coli, lane 3- test strain E.coli ST131 clade C (the resultant image is a product of time-averaged data). (PNG) [file pone.0227725.s003.png]
