# Supplementary material for: Detection and characterization of ESBL-producing Enterobacteriaceae from the gut of healthy chickens, Gallus gallus domesticus in rural Nepal: Dominance of CTX-M-15-non-ST131 Escherichia coli clones
Source: PLoS One. 2020 May 29;15(5):e0227725. doi: 10.1371/journal.pone.0227725 (PMC7259619; doi:10.1371/journal.pone.0227725)
Supplement: S1 Table — (DOCX) [file pone.0227725.s004.docx]

**Supplement file: Table S4: List of primers**

| **Sl. no** | **PCR name** | **β-lactamase(s) targeted** | **Sequence (50 –30)** | **Length**  **(bases)** | **Amplicon**  **size (bp)** | **Primer**  **concentration**  **(pmol/mL)** | **Reference** |
| --- | --- | --- | --- | --- | --- | --- | --- |
| **1** | **Multiplex I**  TEM, SHV and  OXA-1-like | **TEM variants** including TEM-1 and  TEM-2 | F-CATTTCCGTGTCGCCCTTATTC  R-CGTTCATCCATAGTTGCCTGAC | 22  22 | 800 | 0.4  0.4 | 24 |
|  |  | **SHV variants** including SHV-1 | F-AGCCGCTTGAGCAAATTAAAC  R-ATCCCGCAGATAAATCACCAC | 21  21 | 713 | 0.4  0.4 |  |
|  |  | **OXA-1, OXA-4 and OXA-30** | F-GGCACCAGATTCAACTTTCAAG  R-GACCCCAAGTTTCCTGTAAGTG | 22  22 | 564 | 0.4  0.4 |  |
| **2** | **Multiplex II**  CTX-M group 1,  group 2 and group 9 | **Variants of CTX-M group 1** including CTX-M-1, CTX-M-3 and CTX-M-15 | F-TTAGGAARTGTGCCGCTGYAb  R-CGATATCGTTGGTGGTRCCAT^b^ | 20  21 | 688 | 0.4  0.2 | 24 |
|  |  | **Variants of CTX-M group 2** including CTX-M-2 | F-CGTTAACGGCACGATGAC  R-CGATATCGTTGGTGGTRCCAT^b^ | 18  21 | 404 | 0.2  0.2 |  |
|  |  | **Variants of CTX-M group 9 including CTX-M-9 and CTX-M-14** | F-TCAAGCCTGCCGATCTGGT  R-TGATTCTCGCCGCTGAAG | 19  18 | 561 | 0.4  0.4 |  |
| **3** | **Singleplex** | **CTX-M-15** | F-CACACGTGGAATTTAGGGACT  R-GCCGTCTAAGGCGATAAACA | 21  20 | 996 | 0.1  0.1 | 25 |
| **4** | **Multiplex III AmpC genes**  FOX, MOX, DHA, and CIT | **FOX-1 to FOX-5** | F-CTACAGTGCGGGTGGTTT  R- CTATTTGCGGCCAGGTGA | 18  18 | 162 | 0.5  0.5 | 24 |
|  |  | **MOX-1, MOX-2, CMY-1, CMY-8 to CMY-11 and CMY-19** | F- GCAACAACGACAATCCATCCT  R- GGGATAGGCGTAACTCTCCCAA | 21  22 | 295 | 0.2  0.2 |  |
|  |  | **DHA-1 and DHA-2** | F- TGATGGCACAGCAGGATATTC  R- GCTTTGACTCTTTCGGTATTCG | 21  22 | 997 | 0.5  0.5 |  |
|  |  | **LAT-1 to LAT-3, BIL-1, CMY-2 to CMY-7, CMY-12 to CMY-18 and CMY-21 to CMY-23** | F-CGAAGAGGCAATGACCAGAC  R- ACGGACAGGGTTAGGATAGT | 20  20 | 538 | 0.2  0.2 |  |
| **5** | **Multiplex IV ST131 clade** | **ST131** | F-AGCAACGATATTTGCCCATT  R-GGCGATAACAGTACGCCATT | 20  20 | 580 | 0.15  0.15 | 26 |
|  |  | **Clade A** | F-TGACGGGACGTGAGCAAATTA  R-AGTCAGACCTAGCCACCCTT | 21  20 | 707 | 0.15  0.15 |  |
|  |  | **Clade B** | F-CAACGTTGAAGCAGTGTATGAG  R-TGACAATCGACGGCTTTAGA | 22  20 | 442 | 0.08  0.08 |  |
|  |  | **Clade C** | F-CGCTGGCCAGTTATCTGAAAT  R- CCTTTCACCAACTGGGTTACT | 21  21 | 103 | 0.2  0.2 |  |

**Legend**: **^b^Y**=T or C; **R**=A or G.
