# Supplementary material for: Detection and characterization of ESBL-producing Enterobacteriaceae from the gut of healthy chickens, Gallus gallus domesticus in rural Nepal: Dominance of CTX-M-15-non-ST131 Escherichia coli clones
Source: PLoS One. 2020 May 29;15(5):e0227725. doi: 10.1371/journal.pone.0227725 (PMC7259619; doi:10.1371/journal.pone.0227725)
Supplement: S2 Table — (DOCX) [file pone.0227725.s005.docx]

**Table S6: Summary of β-lactamase genes identified in 38 ESBL producing isolates**

| **ESBL genotypes** | **Number of isolates (38)** | | | |
| --- | --- | --- | --- | --- |
|  | **Backyard Chickens** | | **Commercial Chickens (broilers)** | |
|  | **N=22** | | **N=16** | |
| **Single Gene** |  | |  | |
| *bla*_TEM-29_ | 00(00) | 17(77.3) | 1 (6.3) | 13(81.3) |
| *bla*_SHV_ | 00(00) |  | 00(00) |  |
| *bla*_OXA-1_ | 00(00) |  | 00(00) |  |
| *bla*_CTX-M-15_ | 17(77.3) |  | 10(62.5) |  |
| *bla*_CTX –M-2_ | 0(00) |  | 1(6.3) |  |
| *bla*_CTX-M-14_ | 00(00) |  | 1(6.3) |  |
| **Multiple genes** |  |  |  |  |
| *bla*_TEM-1_+ *bla*_SHV-1_+ *bla*_CTX-M-15_ | 1(4.6) | 5(22.7) | 1(6.3) | 3(18.8) |
| *bla*_TEM-1_ + *bla*_CTX-M-15_ | 2(9.1) |  | 1(6.3) |  |
| *bla*_TEM-1_ + *bla*_SHV-1_ + *bla*_OXA-1_+ *bla*_CTX-M-15_ | 1(4.6) |  | 0 (00) |  |
| *bla*_OXA-1_+ *bla*_CTX-M-15_ | 00 (00) |  | 1(6.3) |  |
| *bla*_TEM-2_+ *bla*_OXA-1_+ *bla*_CTX-M-15_ | 1(4.6) |  | 00 (00) |  |
