# Supplementary material for: Detection and characterization of ESBL-producing Enterobacteriaceae from the gut of healthy chickens, Gallus gallus domesticus in rural Nepal: Dominance of CTX-M-15-non-ST131 Escherichia coli clones
Source: PLoS One. 2020 May 29;15(5):e0227725. doi: 10.1371/journal.pone.0227725 (PMC7259619; doi:10.1371/journal.pone.0227725)
Supplement: S1 File — (DOCX) [file pone.0227725.s006.docx]

**Questionnaires for Rectal swab/stool specimen collection in Poultry**

**Sl. No: VDC No.: House no.: House owner Name and Address:**

| Type of farming | Commercial | Household |
| --- | --- | --- |
| Place |  |  |
| Socioeconomic status |  |  |
| Breed type |  |  |
| Age of bird |  |  |
| Farm size |  |  |
| Feed type | Commercial | Organic |
| Any known infection or signs of infection |  |  |
| Treatment/ Antibiotic use |  |  |
| No. of birds sampled |  |  |
| Date and time of sample collection |  |  |
| Regional veterinarian remarks |  |  |
|  |  |  |

**Remarks:**

**Signature of field work candidate:**

**Signature of house or farm owner:**

| **To be filled by Research investigator in Lab:**   1. Total no. of specimens received : 2. Date and time; sample received and processed: 3. Remarks: 4. Signature: |
| --- |
